# Characterization at nucleotide resolution of the homogeneously staining region sites of insertion in two cancer cell lines

**DOI:** 10.1093/nar/gkt566

**Published:** 2013-07-02

**Authors:** Anne Gibaud, Nicolas Vogt, Olivier Brison, Michelle Debatisse, Bernard Malfoy

**Affiliations:** ^1^Institut Curie, Centre de Recherche, ^2^CNRS, UMR3244 and ^3^UPMC, 26 Rue d'Ulm, F-75248 Paris, France

## Abstract

The mechanisms of formation of intrachromosomal amplifications in tumours are still poorly understood. By using quantitative polymerase chain reaction, DNA sequencing, chromosome walking, *in situ* hybridization on metaphase chromosomes and whole-genome analysis, we studied two cancer cell lines containing an *MYC* oncogene amplification with acquired copies ectopically inserted in rearranged chromosomes 17. These intrachromosomal amplifications result from the integration of extrachromosomal DNA molecules. Replication stress could explain the formation of the double-strand breaks involved in their insertion and in the rearrangements of the targeted chromosomes. The sequences of the junctions indicate that homologous recombination was not involved in their formation and support a non-homologous end-joining process. The replication stress-inducible common fragile sites present in the amplicons may have driven the intrachromosomal amplifications. Mechanisms associating break-fusion-bridge cycles and/or chromosome fragmentation may have led to the formation of the uncovered complex structures. To our knowledge, this is the first characterization of an intrachromosomal amplification site at nucleotide resolution.

## INTRODUCTION

The relationships between the two forms of gene amplification found in tumours, the intrachromosomal homogeneously staining regions (hsr) and the extrachromosomal DNA molecules, named double minutes (dmins), are not well established. Dmins may result from the circularization of a single fragment or from the fusion of several syntenic or non-syntenic DNA fragments formed after chromosome fragmentation/pulverization (1–6). Two models are proposed to explain the formation of hsr. In the intra-chromosomal breakage-fusion-bridge (BFB) model, multiple cycles of BFB lead to the amplification of a segment at the site that bears the locus in non-amplified cells [reviewed in (7)]. This mechanism was demonstrated *in vitro* experiments (8–11) and explains the structure of hsr found in tumours (12–16). In the second model, dmins may fuse and eventually integrate into a chromosome, giving rise to hsr (17). The fusion of extrachromosomal molecules followed by chromosome reintegration has been observed in several systems (10,11,18–20). Hence, data are now available, suggesting that fusion and reintegration constitute a pathway for the evolution of extrachromosomal elements that may account for the frequent observation of ectopic hsr in cells of human cancers. However, the site of insertion of an hsr has never been characterized at the nucleotide resolution.

We analysed here the structure of rearranged chromosomes 17 bearing an hsr harbouring an amplified *MYC* region in two human cancer cell lines. The formation of the double-strand breaks (DSBs) involved in the hsr insertion and the chromosome rearrangements could result from replication stress. The sequences of the junctions indicate that homologous recombination was not involved in their formation. Mechanisms associating BFB cycles and/or chromosome fragmentation may have led to the formation of the complex chromosome structures characterized in these tumour cells.

## MATERIALS AND METHODS

### Biological material

The glioblastoma (tumour 11) was collected at the Hôpital de la Salpêtrière (Paris). Informed consent was obtained from the patient. The tumour was grown as a xenograft in athymic mice, and a cell line (GBM11) was established at passage 5. The human colorectal carcinoma cell lines SW613-3 and SW613-Tu1 were as previously described (21).

### Cell cultures and fluorescence *in situ* hybridization

The cell lines were grown as previously described (21). Metaphase spreads were hybridized with bacterial artificial chromosomes (BAC) and fosmid (Chori-BACPAC Resources) or chromosome-specific painting probes (Kreatech Diagnostics) as previously described (Supplementary Materials and Methods) (1). For the induction of common fragile sites, cells were treated with aphidicolin (Sigma, 0.6 µM) for 16 h before metaphase spreading. The percentage of breaks in the hsr was calculated after recording the breaks in ∼300 metaphases (22).

### Amplicon analysis

The structure of the amplicons and the location of the hsr were determined using molecular and fluorescence *in situ* hybridization (FISH) approaches as previously described (Supplementary Materials and Methods) (1). Sequence data used in this work refer to the human genome sequence (released February 2009) available at the UCSC Genome Bioinformatics site (http://genome.ucsc.edu/) (23).

### DNA copy number determination

Copy number alterations were detected as previously described, using Affymetrix SNP-array 6.0 (Supplementary Materials and Methods) (3). Data were normalized, analysed and visualized using Partek Genomic Suite version 6.6 (Partek Inc., St Louis, MO, USA) and the Genome Alteration Print (GAP) software (24). Microarray data were deposited in the ArrayExpress database (accession: GBM11, E-MEXP-3285; SW613-Tu1, E-MEXP-3278; SW613-3, E-MEXP-3277).

## RESULTS

### Cell line GBM11

**Figure 1.**
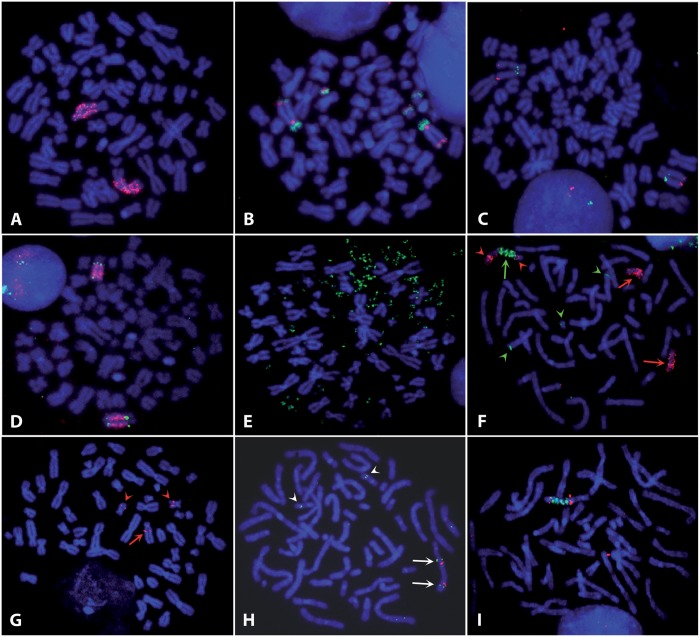
FISH chromosome analysis. (**A**) GBM11. Hybridization of the BAC RP11-1136L8 containing *MYC* (red) on the two copies of the hsr localized in 17p11. (**B**) GBM11. Co-hybridization of paintings of the short (red) and long (green) arms of chromosome 17. Three small rearranged chromosomes were labelled in addition to the two chromosomes bearing the hsr. (**C**) GBM11. Localization of the hsr. Two abbreviation added in M&M of chromosome 17, RP11-76J15 (red) at position 17 056 286–17 231 335 and RP11-160E2 (green) at position 18 923 089–19 081 309 hybridized, respectively, in the telomeric and centromeric positions of the hsr. (**D**) GBM11. Hybridization of CTD-2277H24 (green) both sides of the hsr localized the insertion site in the 17 661 837–17 794 698 region of the chromosome 17. The hsr was labelled in red with BAC RP11-1136L8 (**E**) SW613-3. FISH labelling of the dmins by the BAC RP11-1136L8 containing *MYC* (green). (**F**) SW613-Tu1. Co-hybridization of a chromosome 17 painting (red) and of the BAC RP11-1136L8 containing *MYC* (green). Cells contains two normal chromosomes 17 (red arrows) in addition to the rearranged chromosome (green arrow). The distal part of the rearranged chromosome came from another chromosome, but chromosome 17 was present on both sides of the hsr (red arrowheads). The *MYC* regions on the two normal chromosomes 8 were labelled by the probe (green arrowheads). (**G**) SW613-Tu1. Localization of the hsr on chromosome 17. FISH of BAC RP11-9G4 (position 53 239 436–53 421 980). The probe was in a centromeric position of the hsr (red arrow). The two normal chromosomes 17 were also labelled (red arrowheads). (**H**) SW613-Tu1. Localization of the hsr on chromosome 17. FISH of BACs RP11-260G21 (red, position 64 038 912–64 219 416) and RP11-162H19 (green, position 53 627 465–53 781 044). Probes hybridized symmetrically on both sides of the hsr (arrows). The two normal chromosomes 17 were also labelled (arrowheads). (**I**) SW613-Tu1. Localization of the hsr on chromosome 17. FISH of BAC RP11-1119G22 (red, position 61 934 712–62 106 824). The labelling is observed on both sides of the hsr. This BAC was the more distal BAC hybridizing on the rearranged chromosome, indicating that the insertion site was in this region. The hsr was labelled in green by the BAC RP11-1136L8-containing *MYC*. Chromosomes were stained in blue with 4′,6-diamidino-2-phenylindoli (DAPI).

In glioblastoma cell line GBM11, the 8q24 chromosome region containing the *MYC* gene was amplified as an hsr localized in 17p11 (25). Clones exhibiting dmins were observed in the multiclonal fresh tumour (26). FISH experiments using an *MYC* probe revealed the presence of two chromosomes bearing the hsr in hypo-tetraploid cells (64–68 chromosomes), indicating that the formation of the hsr preceded tetraploidization (Figure 1A). Painting of chromosome 17 arms (Figure 1B) confirmed the insertion of the hsr in the short arm. In addition, a segment from the long arm was translocated to the end of the short arm. Three small rearranged chromosomes containing segments from chromosome 17 short and long arms are present in the cells, but no normal chromosome 17 is present.

**Figure 2.**
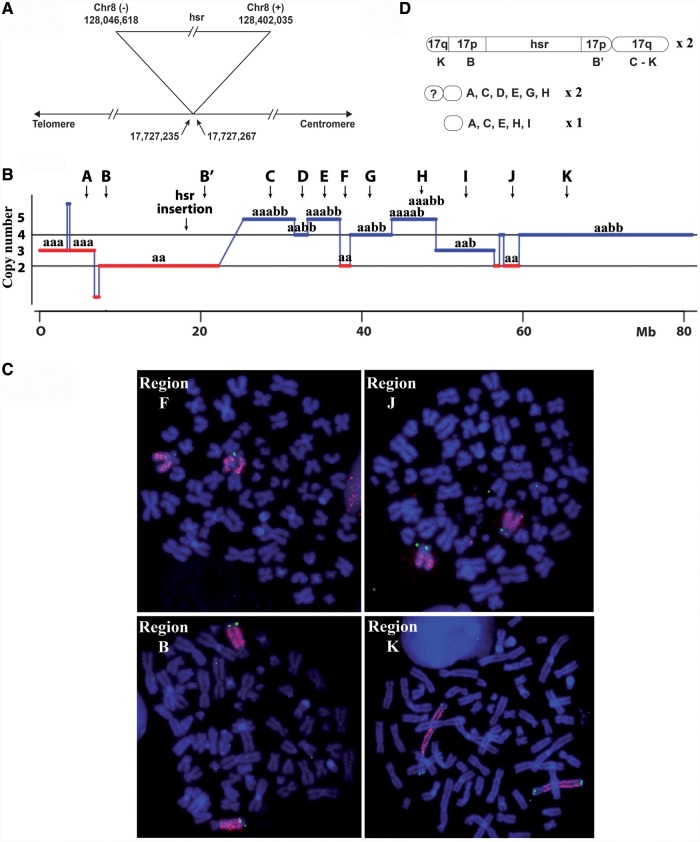
Rearranged chromosomes 17 in GBM11. Full data are available in Supplementary Figure S2 and Supplementary Data S2. (**A**) Site of insertion of the hsr. The insertion was associated with a deletion of 31 bp in the chromosome 17. The junction was between two sequences of the hsr in opposite orientation. The positions of the junctions in the sequences are indicated in base pairs. (**B**) Synthesis of the copy number and allelic variation data from SNP analysis. a and b: alleles; A–K: chromosome regions with different copy numbers and allelic compositions, B and B′ indicate the regions each side of the hsr; arrows: position of the BAC used for FISH. (**C**) FISH of BACs localized in selected regions defined in B (FISH of BACs for all regions are presented in Supplementary Data S2). Segments B, F, J and K were present only in the chromosome bearing the hsr, segment K was duplicated and one copy was translocated to the short arm. Chromosomes were stained in blue with DAPI. (**D**) Schematic representation of the chromosome containing the hsr and of the three small rearranged chromosomes. The A–K segments are defined in D, ?: chromosome segment of unknown origin.

Quantitative polymerase chain reaction and chromosome walking analysis showed that the amplicon comprises a single 1.2-Mb fragment amplified ∼100-fold. The two ends of the original fragment were fused, suggesting that the hsr resulted from the integration of extrachromosomal circular DNA molecules (Supplementary Figure S1). The fusion took place in a 9-bp sequence present at both ends, indicating a microhomology-based non-homologous end-joining mechanism (NHEJ) (1). FISH experiments carried out with probes localized in the 17p11 cytoband were used to map the position of the hsr (Figure 1C) and to find a BAC (CTD-2277H24) overlapping the site of insertion (Figure 1D). By counting the frequency of FISH spots located on both sides of the hsr for a series of overlapping fosmids covering the sequence of this BAC, it was possible to localize the site of insertion in a region of ∼30 kb (17.714–17.744 Mb, Supplementary Data S1). The exact position of the hsr was localized between positions 17 727 235 and 17 727 267 by chromosome walking, corresponding to a deletion of 31 bp in chromosome 17 sequence (Figure 2A and Supplementary Figure S2). Telomeric and centromeric junctions with the *MYC* amplicon are at positions 128 402 035 and 128 046 618 of the amplicon, respectively, the two sequences being in opposite orientation (Figure 2A and Supplementary Figure S2). No sequence homologies were found between the chromosome 8 and 17 sequences involved in the insertion (Supplementary Figure S2 and data not shown).

The organization of the rearranged chromosomes 17 was studied by single-nucleotide polymorphism (SNP) analysis and by FISH (Figure 1B and 2B–D, details of the results are presented in Supplementary Data S2). The copy number profile of the short arm shows no rearrangement in the region containing the hsr (region B-B′, Figure 2B). Thus, the hsr was inserted without modification of the local chromosome organization. FISH experiments with BACs localized in the regions C–K showed that the long arm of the two chromosomes 17 bearing the hsr is not rearranged (Supplementary Data S2 and Figure 2D). The regions F and J of the long arm and the region B-B′ of the short arm (Figure 2C and D) were found only in the chromosomes containing the hsr. These regions are homozygous (Figure 2B), confirming that the two chromosomes containing the hsr result from endoreplication. The K region, corresponding to the distal part of the chromosome 17 long arm, is also present only in the chromosomes containing the hsr (Figure 2C and D). Each chromosome has two copies of this region, one being translocated to the short arm where it replaces the distal A region now found only in the small rearranged chromosomes (Figure 2C and D and Supplementary Data S2). The K region is heterozygous (Figure 2B), suggesting that the translocated fragment came from the second chromosome 17 of the initial cell. The heterozygosity of the other segments indicates that the three small rearranged chromosomes also contain segments from this chromosome. Thus, the insertion of the hsr in GBM11 caused few perturbations in the targeted sequence, but other regions of both chromosomes 17 underwent structural rearrangements.

### Cell lines SW613-3 and SW613-Tu1

These human colorectal carcinoma cell lines were established from tumour induced in mice by the SW613-S cell line, which contains the amplification of *MYC* in the form of dmins (21). In SW613-3, the amplified *MYC* copies remained in dmins (Figure 1E), and dmins containing a segment from the 14q24 cytoband was also detected [(21) and data not shown]. In SW613-Tu1, the amplification of *MYC* was observed in the form of one hsr located at 17q24, whereas the 14q24 locus amplification was not detected [Figure 1F and data not shown; (21)]. The amplification of the 14q24 region in SW613-3 was not further analysed.

In both cell lines, the *MYC* amplicon is constituted of the same 3.1-Mb segment, amplified ∼15-fold in SW613-3 and 30-fold in SW613-Tu1 (Supplementary Figure S3 and data not shown). The absence of homology between the sequences of the fused ends and the presence of a single common base at the junction suggest an NHEJ mechanism during the formation of the initial extrachromosomal circular amplicon (1).

**Figure 3.**
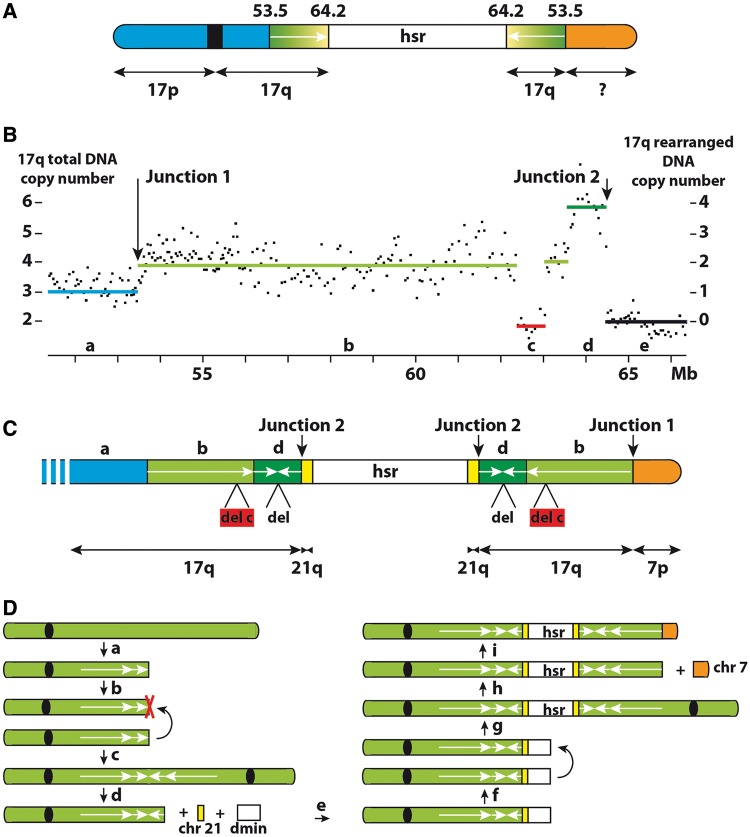
SW613-Tu1. Structure of the chromosome 17 bearing the hsr. (**A**) Schematic representation deduced from FISH analysis. The region 53.5–64.2 Mb is duplicated on both sides of the hsr and is in head-to-head orientation (arrows). (**B**) SNP profile of chromosome 17q in the region of the hsr insertion. The total copy number is indicated on the left, and the number of copies present in the chromosome bearing the hsr (after deduction of the two copies corresponding to the normal chromosomes) is indicated on the right. a–e: regions with different copy numbers. (**C**) Final schematic representation of the site of insertion. Lengths of the regions are not to scale. Regions b, c and d and junctions 1 and 2 are defined in B. The green regions both sides of the hsr are in head-to-head orientation (region b) and contain a deletion of 0.65 Mb (region c). An additional head-to-head duplication of 0.84 Mb is present near the hsr (region c) with a deletion of 1.6 kb at the junction (del). A segment of 156 bp from chromosome 21 is inserted between the chromosome 17 segment and the hsr. The distal part of the chromosome is constituted by the end of the short arm of chromosome 7. (**D**) Model of formation of the rearranged chromosome. A first double-strand chromosome break generated a free DNA end (a), and after replication, two free DNA ends were present on the sister chromatids (b) The sister chromatids then fused and a dicentric chromosome containing the head-to-head duplication was formed (c), red cross: deletion. After a break in the dicentric chromosome during mitosis, a chromosome 21 fragment and *MYC* amplicons were associated (d), and at the end of the process, a double-strand break was formed in the inserted amplified segment (e). At the following cell division (f), the fusion of the broken sister chromatids generated a new dicentric chromosome with the symmetric regions centred on the hsr (g). After a new break in the chromosome 17 (h), the BFB cycles were ended by healing the break with the distal region of the chromosome 7 short arm (i).

Chromosome 17 FISH painting showed that two normal chromosomes are present in pseudodiploid (56–58 chromosomes) SW613-Tu1 cells, in addition to the rearranged chromosome containing the hsr (Figure 1F). The terminal region of this chromosome underwent a translocation with an other chromosome fragment, but sequences from chromosome 17 are present on both sides of the hsr (Figure 1F). A series of BACs was used to specify the position of the hsr in the 17q24 cytoband. Up to position 53.5 Mb, BAC signals were localized centromeric to the hsr (Figure 1G). Between positions 53.5 and 64.2 Mb, BACs hybridized on both sides of the hsr, yielding symmetrical signals with regard to the hsr (Figure 1H and I), indicative of a duplication of this part of the chromosome. BACs with a location more telomeric than 64.2 Mb did not hybridize to the rearranged chromosome, indicating that the distal part of chromosome 17 was lost and replaced by a segment from another chromosome. A rough schematic representation of the rearranged chromosome 17 was deduced from these data (Figure 3A).

The copy number profile of the region of chromosome 17 containing the hsr, deduced from SNP analyses, allowed us to decipher more precisely its structure (Figure 3B and C). Three copies of chromosome 17 sequences were detected up to 53.5 Mb (region a in Figure 3B), corresponding to two normal and one rearranged chromosome. From 64.2 Mb to the end of the long arm, the two copies present on the normal chromosomes were scored (region e in Figure 3B). The segment extending from positions 53.5 to 62.3 Mb (region b in Figure 3B) is present in four copies, located on the two normal chromosomes and on both sides of the hsr. The distal region of this segment is complex with two copies of the 62.3–63 Mb (c) and 63.6–64.2 Mb (d) regions, lost and gained, respectively.

Starting from the copy number transition regions, the precise structure of the rearranged chromosome was established using chromosome walking. (Supplementary Figure S3). The global structure of the rearranged chromosome 17 was deduced from these data (Figure 3C). The previously unidentified fragment at the end of the rearranged 17q arm proved to be a fragment from chromosome 7 p (junction 1 in Figure 3B). The fusion, confirmed by FISH (Supplementary Figure S4), was located between positions 53 497 490 on chromosome 17 and 23 542 171 on chromosome 7. The deleted region, present in two copies (region c in Figure 3B), is 648 502-bp long and located between positions 62 342 817 and 62 991 400. This region, not deleted in SW613-3 (data not shown), contains several copy number variants (Supplementary Figure S5). The 3′-end of region d (Figure 3B) is associated, at position 64 418 667, with another segment of chromosome 17 starting at position 64 420 335, inserted in inverse orientation. The inverted sequence is 840 682-bp long and ends at position 63 579 653. Thus, a head-to-head structure is present, explaining the doubling in DNA copy number observed in this region (region d in Figure 3B). At the junction, a deletion of 1667 bp in the inverted sequence is present. Finally, the chromosome 17-inverted segment is separated from the hsr by a small segment from the long arm of chromosome 21 (156 bp, positions 31 154 562–31 154 717). The junction with the chromosome 8 amplicon takes place at position 127 519 888. Investigations by polymerase chain reaction failed to detect a deletion at the normal position of the chromosome 21 fragment in SW613-Tu1 cells and showed that the fusion between chromosome 8 and 21 segments is absent in SW613-3 cells (data not shown). FISH experiments revealed the translocation of one of the three chromosomes 21 on another chromosome in SW613-Tu1 cells, but not in SW613-3 cells (Supplementary Figure S4). The fusion took place at the centromeres. SNP experiments did not detect copy number abnormalities in chromosome 21 sequences, indicating the absence of rearrangements during the fusion (data not shown). One Alu-repeated sequence is present in the vicinity of the junctions in the three segments from chromosomes 17, 21 and 8 (Supplementary Figure S6). No sequence homologies that could support a homologous recombination mechanism were found at the junctions (Supplementary Figure S3 and data not shown). Even in the case of chromosome 17, 21 and 8 segments, where fusions took place within or near Alu-repeated sequences, no local homologies were found. Of the five junctions analysed, microhomologies of 1 and 2 bp were found in two cases, one insertion of 2 bp was observed in a single case, and the fusion was blunt in the last two cases. Together, these data suggest an NHEJ mechanism for the formation of the junctions. Thus, the site of insertion of the hsr in SW613-Tu1 was complex, and the targeted chromosome 17 underwent multiple structural rearrangements.

### Common fragile sites in *MYC* amplicons

**Figure 4.**
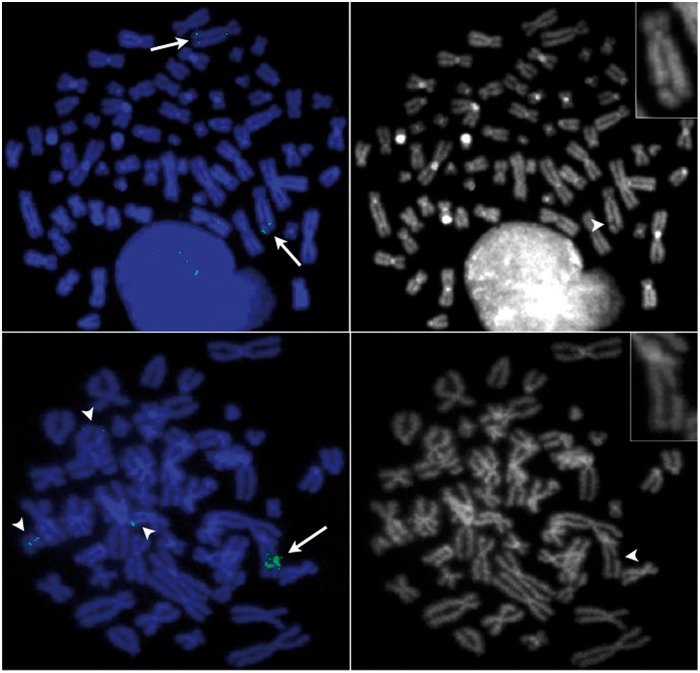
Aphidicolin induction of the common fragile sites FRA8C and FRA8D in the hsr. (**A** and **B**) GBM11. (**C** and **D**) SW613-Tu1. (A and C) hsr were visualized in green (arrows) by hybridization of the BAC RP11-76J15 localized in the telomeric position of the hsr (GBM11) or of the BAC RP11-1136L8 containing the gene *MYC* (SW613-Tu1). FISH signals were also observed on normal or rearranged chromosomes without amplification (arrow heads). Chromosomes were stained in blue with DAPI. (B and D) Reverse DAPI staining, a break was present in the hsr (arrow heads) (in GBM11 the second hsr was not broken). The broken chromosomes were enlarged in the inserts.

The *MYC* oncogene is located between two aphidicolin (APC)-sensitive common fragile sites (CFSs), FRA8C and FRA8D (27), and these CFS regions were amplified in GBM11 and SW613-Tu1 cells. APC induces breaks by slowing down the replication forks in these difficult-to-replicate regions (22,28). The frequency of APC-induced DNA breaks in CFSs varies between individuals and with cell type (29). For FRA8C and FRA8D, data are available for lymphocytes only, and breaks at these loci amount to <1% of the total number of breaks (30). After treatment with APC (Figure 4), breaks in the hsr of GBM11 and of SW613-Tu1 cells represent 30 and 18% of the breaks, respectively. This high rate of breakage indicates that the CFSs present in the *MYC* amplicons are still sensitive to replication stress.

## DISCUSSION

We have analysed at nucleotide resolution the site of insertion of the hsr present in a glioblastoma (GBM11) cell line and a colorectal carcinoma (SW613-Tu1) cell line. In both cases, the oncogene *MYC* was amplified, and the hsr is located at an ectopic position in the short (GBM11) or the long (SW613-Tu1) arm of rearranged chromosome 17. The extrachromosomal amplification of *MYC* was observed in the initial tumour for GBM11 (26) and in a cell line (SW613-3) derived from the same carcinoma as SW613-Tu1. The presence of the same amplicon in SW613-3 and SW613-Tu1 cells indicates that the dmins and the hsr derived from a single initial event. In addition, in both cell lines, the initial amplicons were constituted by a single fragment whose two ends were fused by an NHEJ mechanism, suggesting the initial formation of dmins (1). These data are in good agreement with a mechanism where the first step of the amplification was the formation of dmins from the region containing *MYC* of chromosome 8, which was later integrated into chromosome 17.

### Involvement of the replication stress

Homologies were not found between the sequences involved in the insertion sites of the hsr or in the other rearrangements (Supplementary Figures S2 and S3). This lack of homology precludes homologous recombination mechanisms. The structure of the junctions, where microhomologies of a few base pairs were found in some cases, supports the involvement of an NHEJ process in fusing DSBs.

In cancer cells, replication stress is considered as a main cause of chromosome instability induced by DSBs (31–33). Our data on GBM11 and SW613-Tu1 cell lines support a role for such a stress in the formation of the hsr and of the rearranged chromosomes where they were inserted.

It was reported that in Chinese hamster cells, the replication stress caused by drugs, such as APC, induces breaks in CFSs present in dmins, driving their fusion and their ectopic reintegration into chromosomal CFSs, generating hsr (10). The presence of CFSs inducible by replication stress in the *MYC* amplicons (Figure 4) suggests that a similar mechanism may have existed in GBM11 and SW613-Tu1 cells for the formation of DSBs in the dmins before their integration. In contrast, the association of the sites of insertion with intrachromosomal CSFs is difficult to establish, as the CSF potentially present in these regions (FRA17C in 17p11 and FRA17E in 17q24–25) is poorly mapped (30). However, replication stress may induce numerous breaks across the genome (34), and the breaks targeted by the dmins could occur outside known CFSs.

### Mechanisms of formation of the hsr in GBM11 cells

GBM11 cells contain two identical rearranged chromosomes 17 bearing an hsr (Figure 1A), an indication that hsr formation preceded the endoreplication in these pseudo-tetraploid cells. No normal chromosome 17 is present, but three small marker chromosomes were labelled by chromosome 17 painting (Figure 1B). FISH and SNP experiments showed that various heterozygous fragments from the short and long arms of chromosome 17 are distributed in these rearranged chromosomes (Figure 2B and Supplementary Data S2), indicating that some translocated segments came from the second chromosome 17 initially present in the cell. This complex situation suggests that chromosomes 17 were fragmented at the time of the formation of the rearranged chromosomes. Chromosome fragmentation (also named pulverization) is one of the models proposed to explain the formation of complex dmins in human tumours (3,35,36) and of extensive intrachromosomal rearrangements (chromothripsis) [review in (32,37)]. It has been shown that this chromosome destabilization can be caused by a replication stress experienced by chromosomes trapped in micronuclei (38). Thus, DSBs induced by a replication stress in dmins and in chromosomes 17 trapped in a micronucleus may have initiated the formation of the rearranged chromosomes. However, hsr formation was not the consequence of the fusion of dmins with two fragments from different regions of chromosome 17, as the hsr is inserted in the middle of a non-rearranged region (Figure 2C). The insertion took place in the first exon of the sterol regulatory element-binding transcription factor 1 (*SREBF1*) gene located within the Smith–Magenis syndrome region (39). This region is prone to chromosome rearrangements found in several genetic diseases. However, the site of hsr insertion is not located in one of the low-copy repeats involved in these rearrangements. In the hsr, two sequences in inverse orientation and located >350 kb apart in the initial amplified segment were involved in the fusion (Figure 2A). It is not possible to determine whether this structure is the result of the fusion mechanism or corresponds to a previous rearrangement of the inserted sequence. In contrast, the 31-bp deletion in chromosome 17 is likely because of the insertion mechanism or because of the processing of the DSBs before fusion. However, beyond the involvement of an NHEJ process, the available data do not shed light on the details of the mechanism of insertion.

### Mechanisms of formation of the hsr in SW613-Tu1 cells

In these cells, the hsr is located in the long arm of a single rearranged chromosome 17 (Figures 1F and 3C). We propose that this hsr was formed during BFB cycles undergone by chromosome 17, by the insertion of fused dmins (see earlier in the text). Two BFB cycles can explain the formation of the symmetrical structures present in the rearranged chromosome 17 (Figure 3D). The BFB model predicts that the sequences on either side of the junction should be identical. However, during the fusion of the sister chromatids in the first cycle (b and c in Figure 3D), a deletion of 1.6 kb occurred in one of the chromatids (red cross Figure 3D). Erosion of the chromatids in a few kilo base pairs before fusion was previously documented in similar contexts (40,41). It was proposed that the erosion resulted from single-strand exonuclease digestion of one sister chromatid during an aborted attempt of strand invasion and repair via homologous recombination (40). During the insertion of the hsr (d and e in Figure 3D), the presence of Alu sequences in the vicinity of the junctions may have favoured the association of the segments from chromosomes 8, 17 and 21, but this does not imply that the events were concomitant. The detailed mechanism of junction formation between chromosome 17 and the hsr could not be precisely established, and several BFB cycles with DSBs within the hsr cannot be ruled out. However, *in fine*, a symmetrical organization on both sides of the hsr was obtained (f and g in Figure 3D). The BFB cycles were then ended by healing the break in the chromosome 17 with the chromosome 7 short-arm fragment (i in Figure 3D). In contrast with the classic BFB model that results in an *in loco* intrachromosomal amplification with the formation of a ladder-like structure with a head-to-head organization, in the model proposed here, the region of insertion gained only a few copies.

The presence of the chromosome 21 fragment is likely related to the formation of the rearranged chromosome 21 observed in SW613-TU1 but not in SW613-3 cells (Supplementary Figure S4). However, we failed to detect a deletion at the site of the fragment and its mechanism of formation remains an open question. The origin of the chromosome 7 p fragment seems clearer. In SW613-3 cells, four normal chromosomes 7 are present, whereas in SW613-Tu1 cells, only three normal chromosomes 7 remain in the cells (Supplementary Figure S4). Thus, the distal part of the chromosome 7 p arm, which is fused with the rearranged chromosome 17, likely came from the fragmentation of the lacking chromosome, whose other parts were lost. The deletion encompassing the 62.3–62.9-Mb region of chromosome 17 (Figure 3C) arose before or during the BFB cycles, as it is present on both sides of the hsr. This region contains several known copy-number variants (Supplementary Figure S5), and copy-number variants-like gains and losses can be induced throughout the genome by a replication stress (34). Thus, it can be proposed that a replication stress induced this deletion and the breaks in chromosome 7.

## CONCLUSIONS

In conclusion, we have shown that several mechanisms driven by replication stress, such as BFB cycles and/or chromosome fragmentation, could be involved in the transition between extra- and intrachromosomal amplification, resulting in the formation of ectopic hsr. The presently available data are not sufficiently informative to have a full understanding of the insertion process but rule out homologous recombination and support NHEJ mechanisms. To the best of our knowledge, this work is the first characterization at nucleotide resolution of sites of insertion of hsr. A complete picture of the multiple mechanisms leading to their formation will be possible only after detailed analysis of more cases of various origins.

## ACCESSION NUMBERS

ArrayExpress database. Accession: GBM11: E-MEXP-3285, SW613-Tu1: E-MEXP-3278, SW613-3: E-MEXP-3277.

## SUPPLEMENTARY DATA

Supplementary Data available at NAR Online: Supplementary Figures 1–6, Supplementary Materials and Methods and Supplementary Data 1 and 2.

## FUNDING

Institut Curie; Centre National de la Recherche Scientifique. Funding for open access charge: Institut Curie.

*Conflict of interest statement.* None declared.

## Supplementary Material

Supplementary Data

## References

[1] Vogt N, Lefevre SH, Apiou F, Dutrillaux AM, Cor A, Leuraud P, Poupon MF, Dutrillaux B, Debatisse M, Malfoy B (2004). Molecular structure of double-minute chromosomes bearing amplified copies of the epidermal growth factor receptor gene in gliomas. Proc. Natl Acad. Sci. USA.

[2] Storlazzi CT, Fioretos T, Surace C, Lonoce A, Mastrorilli A, Strombeck B, D'Addabbo P, Iacovelli F, Minervini C, Aventin A (2006). MYC-containing double minutes in hematologic malignancies: evidence in favor of the episome model and exclusion of MYC as the target gene. Hum. Mol. Genet..

[3] Gibaud A, Vogt N, Hadj-Hamou NS, Meyniel JP, Hupe P, Debatisse M, Malfoy B (2010). Extrachromosomal amplification mechanisms in a glioma with amplified sequences from multiple chromosome loci. Hum. Mol. Genet..

[4] Blumrich A, Zapatka M, Brueckner LM, Zheglo D, Schwab M, Savelyeva L (2011). The FRA2C common fragile site maps to the borders of MYCN amplicons in neuroblastoma and is associated with gross chromosomal rearrangements in different cancers. Hum. Mol. Genet..

[5] Storlazzi CT, Lonoce A, Guastadisegni MC, Trombetta D, D'Addabbo P, Daniele G, L'Abbate A, Macchia G, Surace C, Kok K (2010). Gene amplification as double minutes or homogeneously staining regions in solid tumors: origin and structure. Genome Res..

[6] Rausch T, Jones DT, Zapatka M, Stutz AM, Zichner T, Weischenfeldt J, Jager N, Remke M, Shih D, Northcott PA (2012). Genome sequencing of pediatric medulloblastoma links catastrophic DNA rearrangements with TP53 mutations. Cell.

[7] Debatisse M, Malfoy B, Nigg EA (2005). Gene amplification mechanisms. Genome Iinstability in Cancer Development.

[8] Kuo MT, Vyas RC, Jiang LX, Hittelman WN (1994). Chromosome breakage at a major fragile site associated with P-glycoprotein gene amplification in multidrug-resistant CHO cells. Mol. Cell. Biol..

[9] Ma C, Martin S, Trask B, Hamlin JL (1993). Sister chromatid fusion initiates amplification of the dihydrofolate reductase gene in Chinese hamster cells. Genes Dev..

[10] Coquelle A, Toledo F, Stern S, Bieth A, Debatisse M (1998). A new role for hypoxia in tumor progression: induction of fragile site triggering genomic rearrangements and formation of complex DMs and HSRs. Mol. Cell.

[11] Coquelle A, Pipiras E, Toledo F, Buttin G, Debatisse M (1997). Expression of fragile sites triggers intrachromosomal mammalian gene amplification and sets boundaries to early amplicons. Cell.

[12] Ciullo M, Debily MA, Rozier L, Autiero M, Billault A, Mayau V, El Marhomy S, Guardiola J, Bernheim A, Coullin P (2002). Initiation of the breakage-fusion-bridge mechanism through common fragile site activation in human breast cancer cells: the model of PIP gene duplication from a break at FRA7I. Hum. Mol. Genet..

[13] Selvarajah S, Yoshimoto M, Park PC, Maire G, Paderova J, Bayani J, Lim G, Al-Romaih K, Squire JA, Zielenska M (2006). The breakage-fusion-bridge (BFB) cycle as a mechanism for generating genetic heterogeneity in osteosarcoma. Chromosoma.

[14] Reshmi SC, Roychoudhury S, Yu Z, Feingold E, Potter D, Saunders WS, Gollin SM (2007). Inverted duplication pattern in anaphase bridges confirms the breakage-fusion-bridge (BFB) cycle model for 11q13 amplification. Cytogenet. Genome Res..

[15] Vukovic B, Beheshti B, Park P, Lim G, Bayani J, Zielenska M, Squire JA (2007). Correlating breakage-fusion-bridge events with the overall chromosomal instability and in vitro karyotype evolution in prostate cancer. Cytogenet. Genome Res..

[16] Lim G, Karaskova J, Beheshti B, Vukovic B, Bayani J, Selvarajah S, Watson SK, Lam WL, Zielenska M, Squire JA (2005). An integrated mBAND and submegabase resolution tiling set (SMRT) CGH array analysis of focal amplification, microdeletions, and ladder structures consistent with breakage-fusion-bridge cycle events in osteosarcoma. Genes Chromosomes Cancer.

[17] Carroll SM, DeRose ML, Gaudray P, Moore CM, Needham-Vandevanter DR, Von Hoff DD, Wahl GM (1988). Double minute chromosomes can be produced from precursors derived from a chromosomal deletion. Mol. Cell. Biol..

[18] Pipiras E, Coquelle A, Bieth A, Debatisse M (1998). Interstitial deletions and intrachromosomal amplification initiated from a double-strand break targeted to a mammalian chromosome. EMBO J..

[19] Shimizu N, Miura Y, Sakamoto Y, Tsutsui K (2001). Plasmids with a mammalian replication origin and a matrix attachment region initiate the event similar to gene amplification. Cancer Res..

[20] Shimizu N, Hashizume T, Shingaki K, Kawamoto JK (2003). Amplification of plasmids containing a mammalian replication initiation region is mediated by controllable conflict between replication and transcription. Cancer Res..

[21] Guillaud-Bataille M, Brison O, Danglot G, Lavialle C, Raynal B, Lazar V, Dessen P, Bernheim A (2009). Two populations of double minute chromosomes harbor distinct amplicons, the MYC locus at 8q24.2 and a 0.43-Mb region at 14q24.1, in the SW613-S human carcinoma cell line. Cytogenet. Genome Res..

[22] Letessier A, Millot GA, Koundrioukoff S, Lachages AM, Vogt N, Hansen RS, Malfoy B, Brison O, Debatisse M (2011). Cell-type-specific replication initiation programs set fragility of the FRA3B fragile site. Nature.

[23] Kent WJ, Sugnet CW, Furey TS, Roskin KM, Pringle TH, Zahler AM, Haussler D (2002). The human genome browser at UCSC. Genome Res..

[24] Popova T, Manie E, Stoppa-Lyonnet D, Rigaill G, Barillot E, Stern MH (2009). Genome Alteration Print (GAP): a tool to visualize and mine complex cancer genomic profiles obtained by SNP arrays. Genome Biol..

[25] Muleris M, Almeida A, Dutrillaux AM, Pruchon E, Vega F, Delattre JY, Poisson M, Malfoy B, Dutrillaux B (1994). Oncogene amplification in human gliomas: a molecular cytogenetic analysis. Oncogene.

[26] Pruchon E, Chauveinc L, Sabatier L, Dutrillaux AM, Ricoul M, Delattre JY, Vega F, Poisson M, Hor F, Dutrillaux B (1994). A cytogenetic study of 19 recurrent gliomas. Cancer Genet. Cytogenet..

[27] Ferber MJ, Eilers P, Schuuring E, Fenton JA, Fleuren GJ, Kenter G, Szuhai K, Smith DI, Raap AK, Brink AA (2004). Positioning of cervical carcinoma and Burkitt lymphoma translocation breakpoints with respect to the human papillomavirus integration cluster in FRA8C at 8q24.13. Cancer Genet. Cytogenet..

[28] Debatisse M, Le Tallec B, Letessier A, Dutrillaux B, Brison O (2012). Common fragile sites: mechanisms of instability revisited. Trends Genet..

[29] Le Tallec B, Dutrillaux B, Lachages AM, Millot GA, Brison O, Debatisse M (2011). Molecular profiling of common fragile sites in human fibroblasts. Nat. Struct. Mol. Biol..

[30] Mrasek K, Schoder C, Teichmann AC, Behr K, Franze B, Wilhelm K, Blaurock N, Claussen U, Liehr T, Weise A (2010). Global screening and extended nomenclature for 230 aphidicolin-inducible fragile sites, including 61 yet unreported ones. Int. J. Oncol..

[31] Burrell RA, McClelland SE, Endesfelder D, Groth P, Weller MC, Shaikh N, Domingo E, Kanu N, Dewhurst SM, Gronroos E (2013). Replication stress links structural and numerical cancer chromosomal instability. Nature.

[32] Jones MJ, Jallepalli PV (2012). Chromothripsis: chromosomes in crisis. Dev. Cell.

[33] Liu P, Carvalho CM, Hastings PJ, Lupski JR (2012). Mechanisms for recurrent and complex human genomic rearrangements. Curr. Opin. Genet. Dev..

[34] Arlt MF, Mulle JG, Schaibley VM, Ragland RL, Durkin SG, Warren ST, Glover TW (2009). Replication stress induces genome-wide copy number changes in human cells that resemble polymorphic and pathogenic variants. Am. J. Hum. Genet..

[35] Campbell PJ, Stephens PJ, Pleasance ED, O'Meara S, Li H, Santarius T, Stebbings LA, Leroy C, Edkins S, Hardy C (2008). Identification of somatically acquired rearrangements in cancer using genome-wide massively parallel paired-end sequencing. Nat. Genet..

[36] Stephens PJ, Greenman CD, Fu B, Yang F, Bignell GR, Mudie LJ, Pleasance ED, Lau KW, Beare D, Stebbings LA (2011). Massive genomic rearrangement acquired in a single catastrophic event during cancer development. Cell.

[37] Forment JV, Kaidi A, Jackson SP (2012). Chromothripsis and cancer: causes and consequences of chromosome shattering. Nat. Rev. Cancer.

[38] Crasta K, Ganem NJ, Dagher R, Lantermann AB, Ivanova EV, Pan Y, Nezi L, Protopopov A, Chowdhury D, Pellman D (2012). DNA breaks and chromosome pulverization from errors in mitosis. Nature.

[39] Lupski JR, Stankiewicz P (2005). Genomic disorders: molecular mechanisms for rearrangements and conveyed phenotypes. PLoS Genet..

[40] Okuno Y, Hahn PJ, Gilbert DM (2004). Structure of a palindromic amplicon junction implicates microhomology-mediated end joining as a mechanism of sister chromatid fusion during gene amplification. Nucleic Acids Res..

[41] Bignell GR, Santarius T, Pole JC, Butler AP, Perry J, Pleasance E, Greenman C, Menzies A, Taylor S, Edkins S (2007). Architectures of somatic genomic rearrangement in human cancer amplicons at sequence-level resolution. Genome Res..

