# Supplementary material for: Characterization at nucleotide resolution of the homogeneously staining region sites of insertion in two cancer cell lines
Source: Nucleic Acids Res. 2013 Jul 2;41(17):8210–9. doi: 10.1093/nar/gkt566 (PMC3783161; doi:10.1093/nar/gkt566)
Supplement: Supplementary Data [file supp_41_17_8210__index.html]

Characterization at nucleotide resolution of the homogeneously staining region sites of insertion in two cancer cell lines — Characterization at nucleotide resolution of the homogeneously staining region sites of insertion in two cancer cell lines — Supplementary Data 

# Characterization at nucleotide resolution of the homogeneously staining region sites of insertion in two cancer cell lines

## 

files

**Files in this Data Supplement:**

- Supplementary Data - pdf file
