# Supplementary material for: Characterization at nucleotide resolution of the homogeneously staining region sites of insertion in two cancer cell lines
Source: Nucleic Acids Res. 2013 Jul 2;41(17):8210–9. doi: 10.1093/nar/gkt566 (PMC3783161; doi:10.1093/nar/gkt566)
Supplement: Supplementary Data [file supp_gkt566_nar-01302-x-2013-File006.pdf]

Supplementary Material and Methods

Cell cultures and Fluorescent in situ hybridization (FISH).

Cell preparations were obtained after short-term culture (1-2 days) of tumor fragments. Metaphase spreads were hybridized with BAC and fosmid (Chori-BACPAC Resources) Bacterial strains containing BAC were spread on LB agar plates containing 12.5 μg ml^{-1} chloramphenicol and grown overnight at 37 °C. BAC DNA was extracted according to the manufacturer's instructions (NucleoBond Xtra Midi Plus; Macherey-Nagel). Probes were labelled with biotin (BIOT) or digoxigenin (DIG) using the BioPrime DNA labelling system (Invitrogen) and purified on Illustra ProbeQuant G-50 Micro Columns (GE Healthcare). FISH on metaphases was performed using 100 ng of BAC probes. Hybridizations were performed in 10% Dextran Sulfate, 50 % formamide, 2SSC, 1% Tween. Immunodetection was performed by successive incubations in the following reagents: for DIG 1) FITC-conjugated mouse anti-DIG, 2) Alexa-488-conjugated goat anti-mouse; for BIOT 1) Texas-Red-conjugated avidin, 2) biotin-conjugated goat anti-Avidin, 3) Texas Red-conjugated Avidine. Slides were washed three times with PBS solution after each layer. Chromosomes were counterstained with 4′,6-diamidino-2-phenylindole (DAPI) (Vectashield mounting medium for fluorescence with DAPI; Vector Laboratories) and metaphases were observed by fluorescence microscopy. Chromosome-specific painting probes were hybridized using the protocole provided by the manufacturer (Kreatech Diagnostics). Breaks or gaps on chromosomes were observed on metaphase spreads stained DAPI.

Amplicon analysis.

The level of amplification was measured by real-time quantitative PCR using the 7500 Real-Time PCR System and SYBR Green PCR kits (Applied-Biosystems). Amplification levels were calculated using a standard curve constructed with serial dilutions of control DNA amplified in a parallel experiment (DNA from normal lymphocytes). DNAs were extracted from lymphocytes and tumor using Qiagen kits. All measurements were performed at least in duplicate. Primers were selected by using PrimerExpress program (Applied Biosystems). Only primer pairs with efficiency higher than 90% were retain for further experiments. The absence of sequences identical to the PCR target in the rest of the genome was verified by using BLAT. PCR fragments were directly sequenced using Big Dye Terminator Sequencing kits (Applied Biosystems).

Whole-genome DNA Copy number determination.

The Affymetrix Genome-Wide Human SNP Array 6.0 was used to detect copy number alterations. Samples were processed at the Genomic Platform of the Institut Curie following the instructions provided by the manufacturer. Data were normalized, analyzed and visualized using Partek Genomic Suite version 6.6 (Partek, St Louis, MO). Single nucleotide polymorphisms with smoothing values lower and greater than 2 ± 0.28 were considered as gain and loss, respectively.

Data S1

GBM11, localisation of the hsr insertion site in 17p11.

The BAC CTD-2277H24 covers the site of insertion of the hsr (Figure 1D in publication). In order to have to precisely localize the insert, FISH were performed using fosmids overlapping the BAC. When a fosmid overlapped the site of insertion, hybridization spots could be observed both side of the hsr (Figure 1). We assumed that the percentage of spots centromeric or telomeric of the hsr was proportional to the lenght of the fosmid sequence in the corresponding side of the insertion site Figure 2A). For the series of fosmids, the percentage of centromeric spots was plotted as a fonction of their 3' or 5' ends positions. The extrapolation of the curves to a percentage of 0 (3' side) or 100 (5' side) determine the location of the insertion. Based on these data, we defined a region of about 30 kb (17,714,000 - 17,743,000) where the insertion site was expected (Figure 2B). Chromosome walking was performed using a series of primers along this chomosome sequence and it was possible to find the junction between chromosomes 17 and the amplicon (see main text)

A B

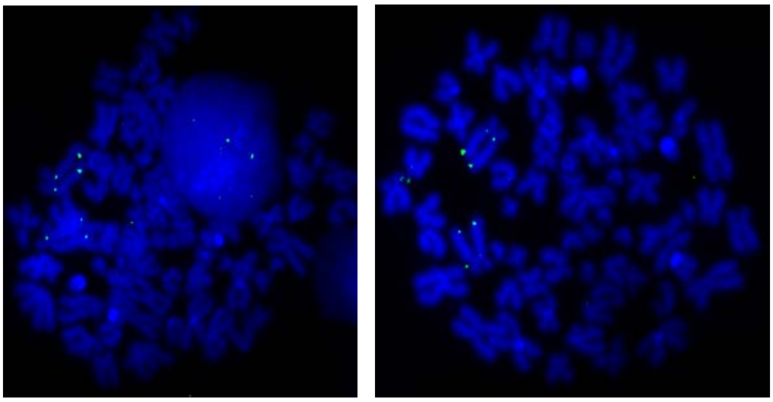

Figure 1. FISH of fosmids overlapping the site of insertion. A: G248P8001A9; B: G248P8413D4.

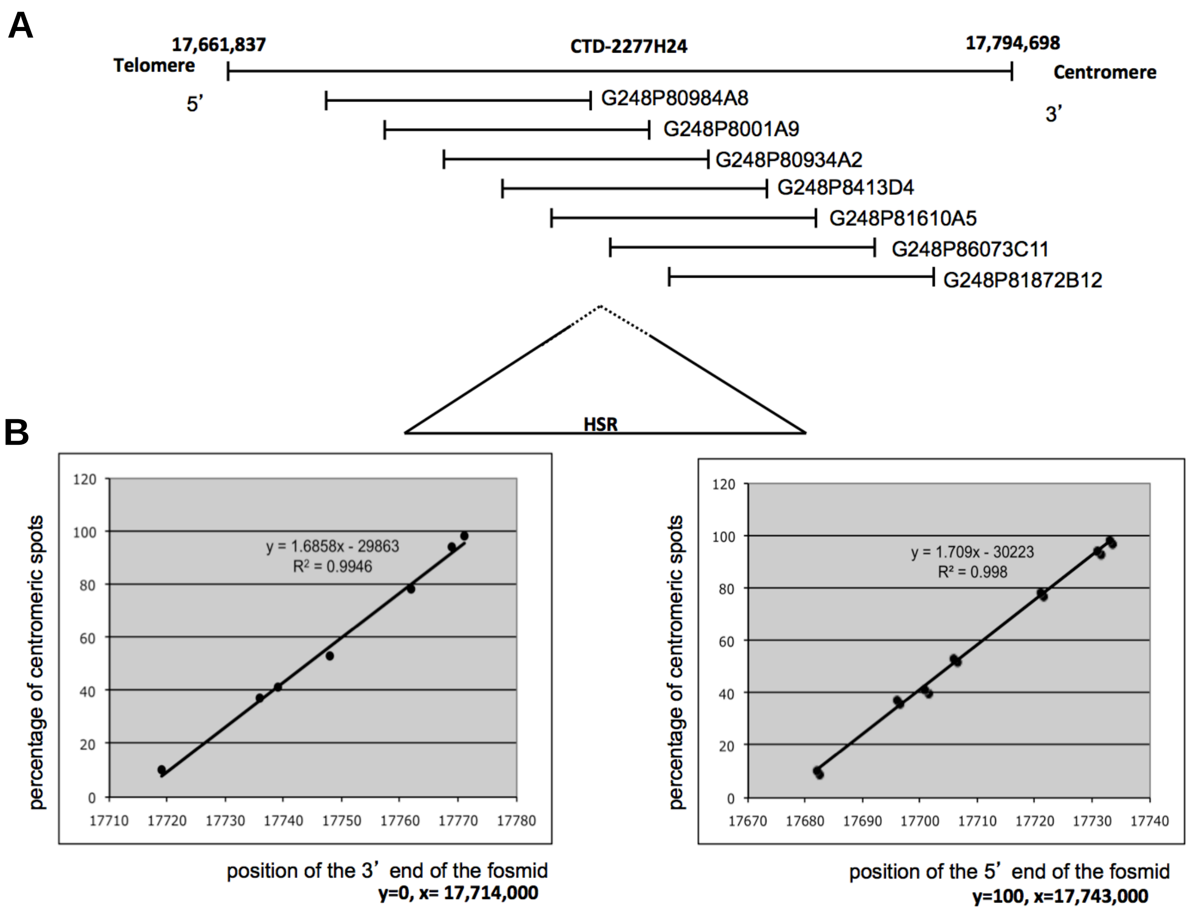

Figure 2. A: Fosmid positions in the hsr insertion region. B. Determination of the insertion region.

Data S2

Structure of chromosome 17 containing the hsr.

FISH of chromosome 17 paintings show that in the chromosomes 17 bearing the hsr a fragment of the long arm was fused at the end of the short arm (Figure 1). In addition, three small marker chromosomes contained segments from the two arms of chromosome 17.

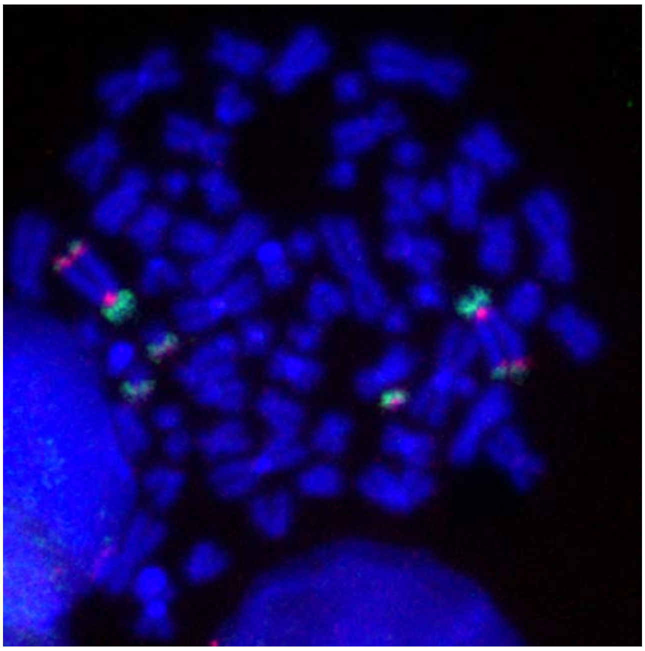

Figure 1. Co-hybridization of paintings of the short (red) and long (green) arms of chromosome 17.

The structure of these chromosomes was studied using SNP analysis. The copy number profile of chromosome 17 sequences deduced from SNP data shows a complex situation (Figure 2A). The allele ratio profile also indicated complex rearrangements (Figure 2B). Eleven regions (A -K) were defined corresponding to the main copy number and allele composition changes (Figure 2C). The boundaries between these regions were not localized at the nucleotide level, but FISH

established their distribution in the rearranged chromosomes. We hybridized BACs (Table 1) localized in the segments A - K (Figure 3). The results are summarized in Table 2. Figure 2D shows the chromosome structure.

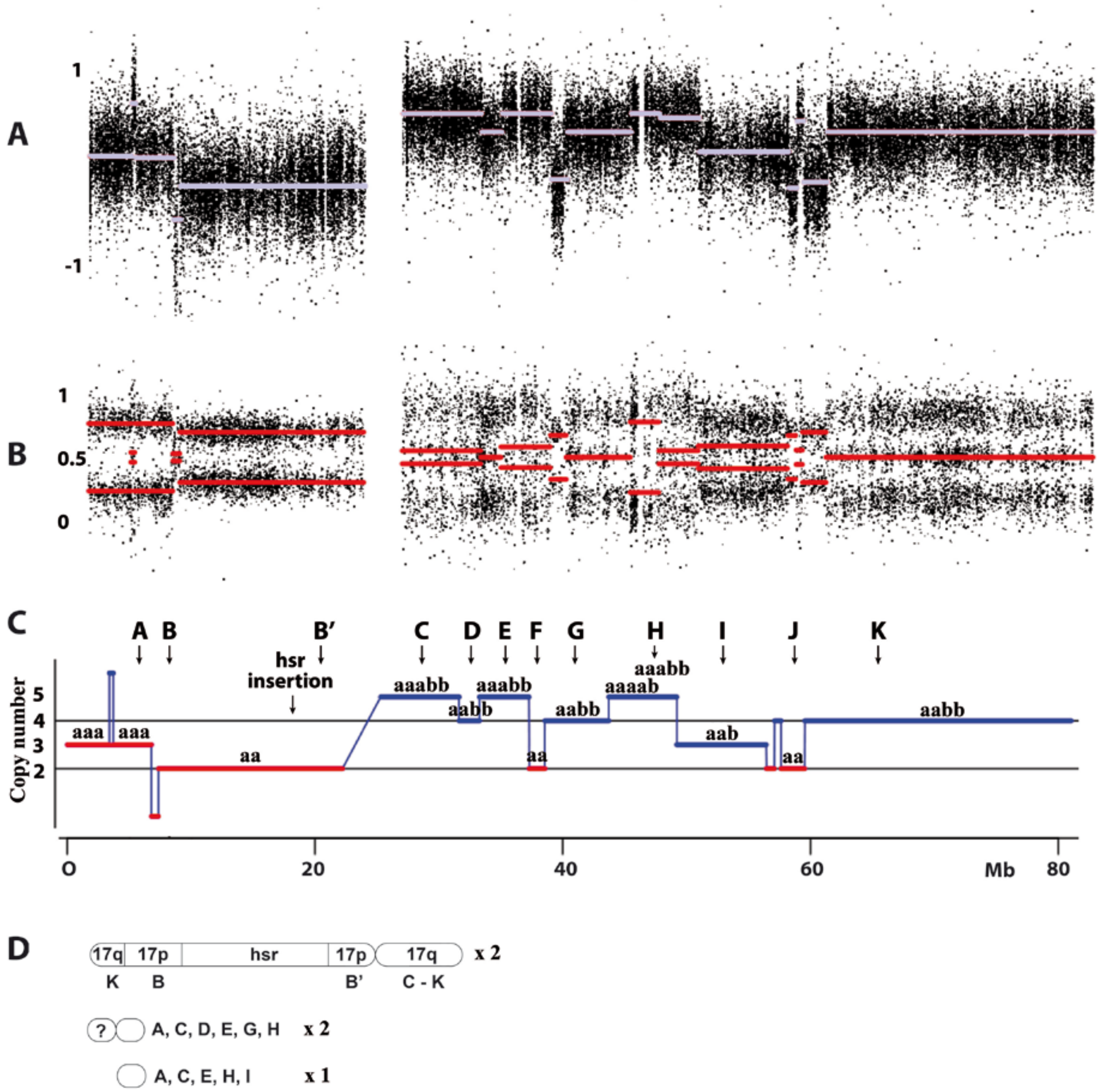

Figure 2. Chromosome 17 SNP array profile and analysis using GAP software (24). **A**. Copy number variations are represented by the log ratio centred at zero. **B.** Allelic imbalances are represented by b-allele frequency. **C**. Synthesis of the copy number and allelic variation data from SNP analysis. a, b: alleles; A - K: chromosome regions with different copy number and allelic composition, B and B' point out the regions each side of the hsr; arrows: position of the BAC used for FISH. **D.** Schematic representation of the chromosome containing the hsr and of the three small rearranged chromosomes. The A-K segments are defined in **C**, ?: chromosome segment of unknown origin.

Segment	BAC	Position ((Mb)
A	RP11-960B9	5.97-6.15
B	RP11-769H22	7.90-8.07
B'	RP11-164N22	20.27-20.44
C	RP11-1095J4	29.60-29.85
D	RP11-60C12	32.12-32.30
E	RP11-904D7	35.16-35.36
F	RP11-353F13	37.52-37.69
G	RP11-209M4	41.69-41.85
H	RP11-111C6	46.57-46.78
I	RP11-800I5	51.75-51.97
J	RP11-1081E4	58.73-58.95
K	RP11-54D5	67.36-67.53

Table 1. Localization on chromosome 17 of the BAC used for FISH

Segment	Copies	Chrom 17 (2 copies)		marker chromosomes		
		p (hsr)	q	1	2	3
A	3	-	-	+	+	+
B	2	+	-	-	-	-
B'	2	+	-	-	-	-
C	5	-	+	+	+	+
D	4	-	+	+	+	-
E	5	-	+	+	+	+
F	2	-	+	-	-	-
G	4	-	+	+	+	-
H	5	-	+	+	+	+
I	3	-	+	-	-	+
J	2	-	+	-	-	-
K	4	+	+	-	-	-

Table 2. Summary of the localization of the chromosome 17 segments

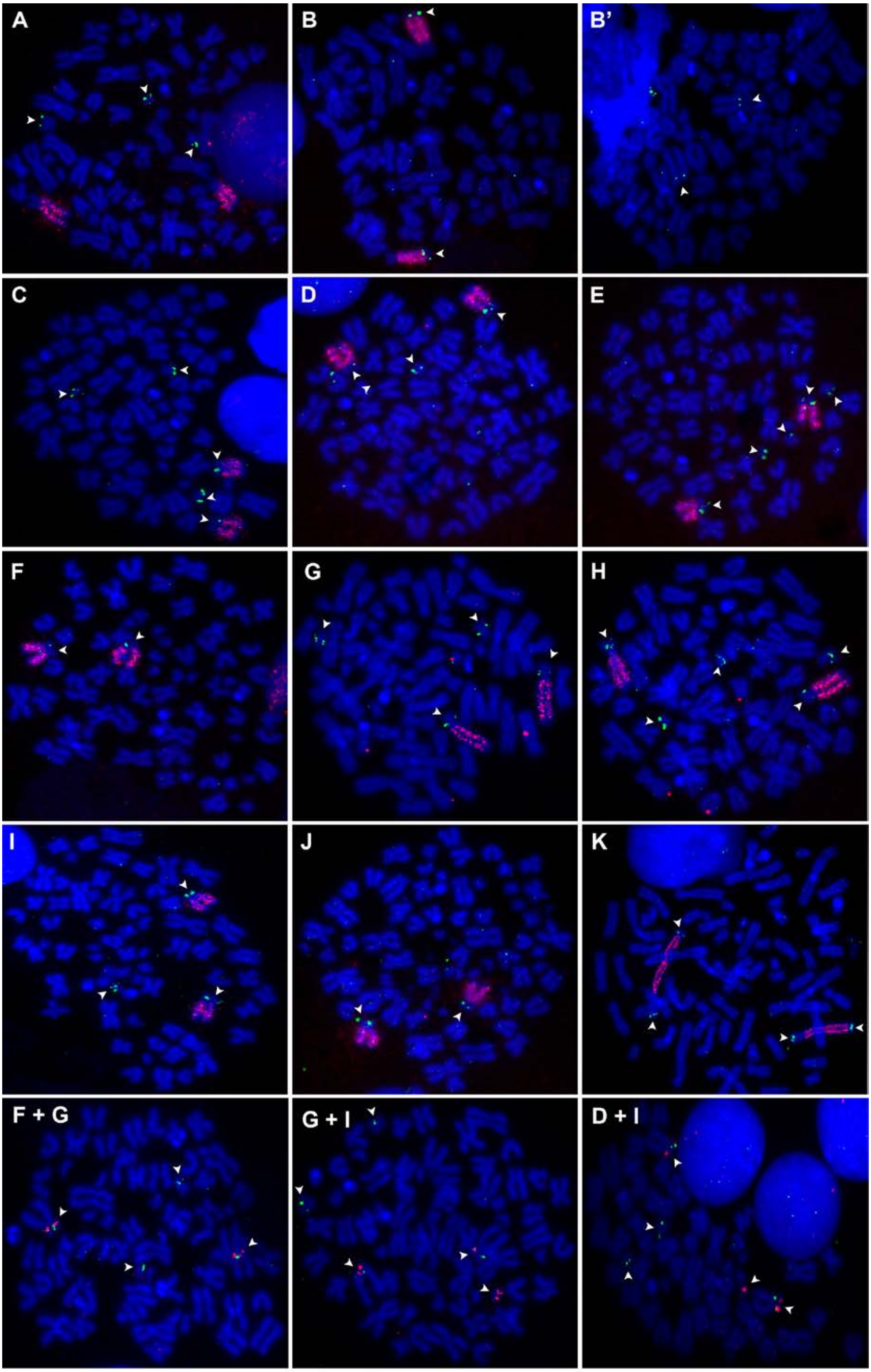
A
B
B'
C
D
E
F
G
H
I
J
K
F + G
G + I
D + I

Figure 3.
FISH of BACs localized along chromosome 17. The positions of the BACs are indicated in Table 1 and in Figure 2C. Table 2 summarizes the data. A - K: chromosome segments defined in Figure 2C. Photos are identified by the letter(s) corresponding to the hybridized BAC. BAC were labelled in green and the hsr was visualized in red using the BAC RP11-1136L8 containing *MYC* in the A-K photos. Arrowheads indicate the BAC hybridization sites.

Segment **A** was found exclusively in the 3 small marker chromosomes.
Segment **B** (two BACs, **B** and **B'**), was present only on the short arm of the 2 chromosomes bearing the hsr (B in telomeric and B' in centromeric positions).
Segments **C**, **E** and **H** were present on the long arm of the chromosome 17 bearing the hsr and on the 3 small marker chromosomes.
Segments **D** and **G** were present on the long arm of the chromosome 17 bearing the hsr and on 2 small marker chromosomes.
Segments **F** and **J** were present only on the long arm of the chromosome 17 bearing the hsr.
Segment **I** was present on the long arm of the chromosome 17 bearing the hsr and on one small marker chromosome.
Segment **K** was present in the telomeric regions of both arms of the 2 chromosomes 17 bearing the hsr.

Co-hybridization of pairs of BACs indicated the composition of the 3 small marker chromosomes (Photos **F+G**, **G+I** and **D+I;** BACs F and I were labelled in red, BACs D and G in green). Two small marker chromosomes had an arm containing the regions A, C, D, E, G and H (the origin of the other arm was not established). The third chromosome (smaller than the other two and likely acrocentric) contained segments A, C, E, H and I.

24 Popova, T., Manie, E., Stoppa-Lyonnet, D., Rigaill, G., Barillot, E. and Stern, M.H. (2009) Genome Alteration Print (GAP): a tool to visualize and mine complex cancer genomic profiles obtained by SNP arrays. *Genome Biol*, **10**, R128.

Figure S1

A

```
AGGGAGGGCTAATGCCTGAGCTCCTGCCCTTTCTATGCAG-TGAGggtcaagatcctcagctagtgtttgagggaactgg
|||||||||||||||||||||||||||||||||||||||| ||||
AGGGAGGGCTAATGCCTGAGCTCCTGCCCTTTCTATGCAGGTGAGttagggtggagtaggtaactggaatgagttagggt
                                    ||||||||||||||||||||||||||||||||||||||||||||
agggtggagcaggtgattggaatgtagtagggtggaGCAGGTGAGTTAGGGTGGAGTAGGTAACTGGAATGAGTTAGGGT
```

B

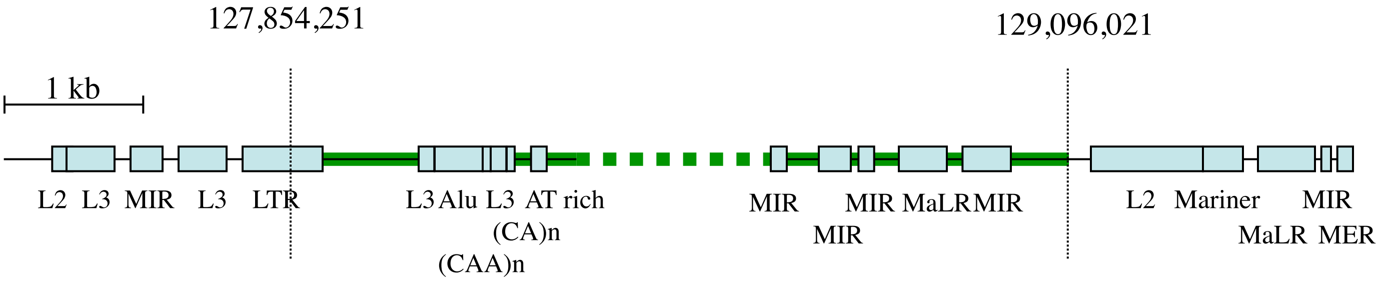

Figure S2

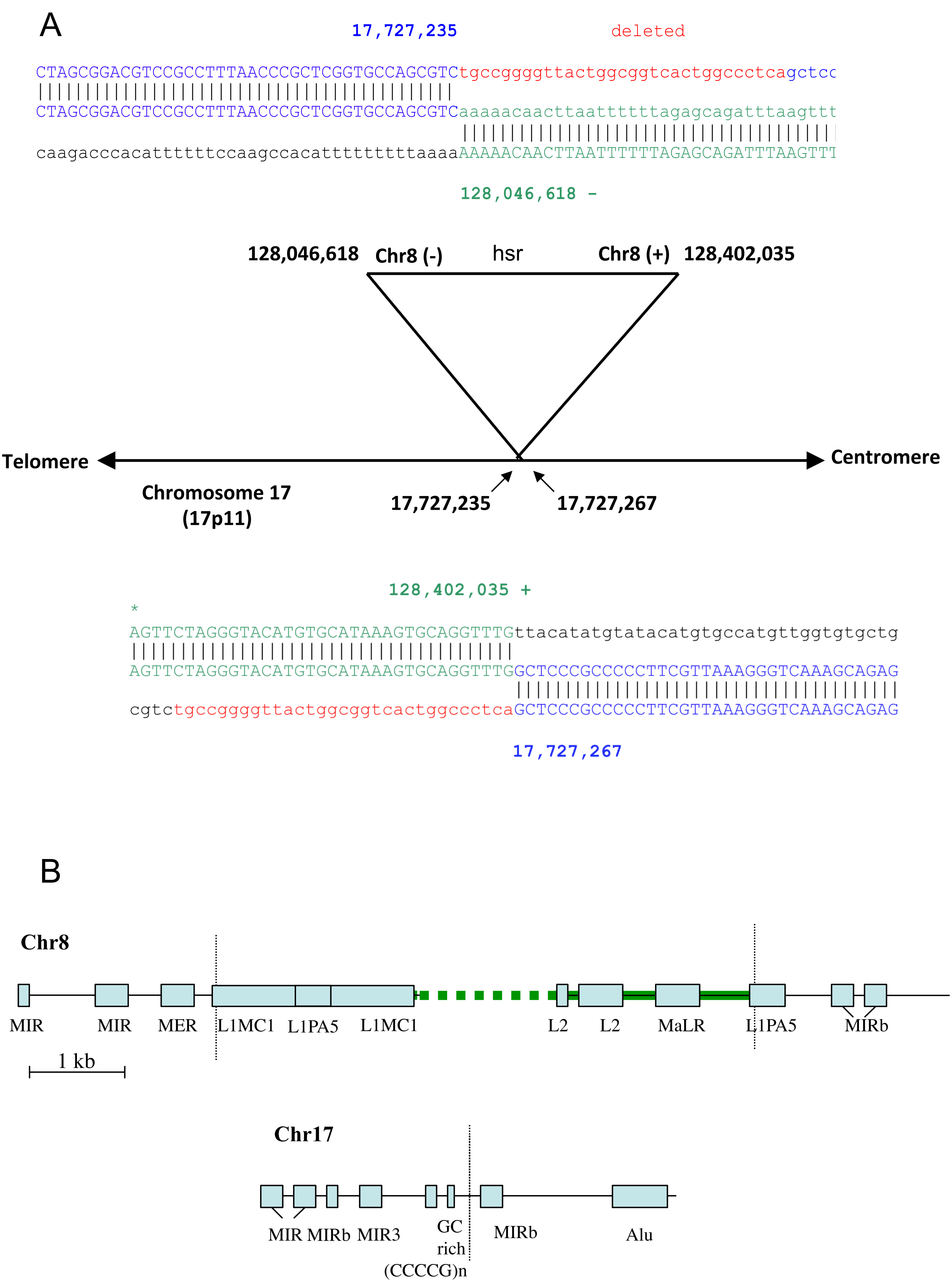
A
17,727,235
deleted
CTAGCGGACGTCCGCCTTTAACCCGCTCGGTGCCAGCGTCtgccggggttactggcggtcactggccctcagctcc
CTAGCGGACGTCCGCCTTTAACCCGCTCGGTGCCAGCGTCaaaaacaacttaatttttagagcagatttaagttt
caagacccacatttttccaagccacattttttttaaaaAAAAACAACTTAATTTTTAGAGCAGATTTAAGTTT
128,046,618 -
128,046,618
Chr8 (-)
hsr
Chr8 (+)
128,402,035
Telomere
Centromere
Chromosome 17
(17p11)
17,727,235
17,727,267
128,402,035 +
*
AGTTCTAGGGTACATGTGCATAAAGTGCAGGTTTGttacatatgtatacatgtgccatgttggtgtgctg
AGTTCTAGGGTACATGTGCATAAAGTGCAGGTTTGGCTCCCGCCCCCTTCGTTAAAGGGTCAAAGCAGAG
cgtctgccggggttactggcggtcactggccctcaGCTCCCGCCCCCTTCGTTAAAGGGTCAAAGCAGAG
17,727,267
B
Chr8
MIR
MIR
MER
L1MC1
L1PA5
L1MC1
L2
L2
MaLR
L1PA5
MIRb
1 kb
Chr17
MIR
MIRb
MIR3
GC
rich
(CCCCG)n
MIRb
Alu

Figure S3

A. Junction of the MYC amplicon

```
                                    129.617.268

ACTTTGTCCTTCAGGAAGATACAATCCTACCATACTGCAATCAAACTGATATTCCAAAACATGACAGACCATGT
|||||||||||||||||||||||||||||||||||||
ACTTTGTCCTTCAGGAAGATACAATCCTACCATACTGTACTTGCCTTTGCTAAATATCAATCATGCATTAGAAA
                                    ||||||||||||||||||||||||||||||||||||||
ATGAAGGGGCTGTTCTTCACTTTACCCAAAGGTCCAGTACTTGCCTTTGCTAAATATCAATCATGCATTAGAAA

                                            126.510.977
```

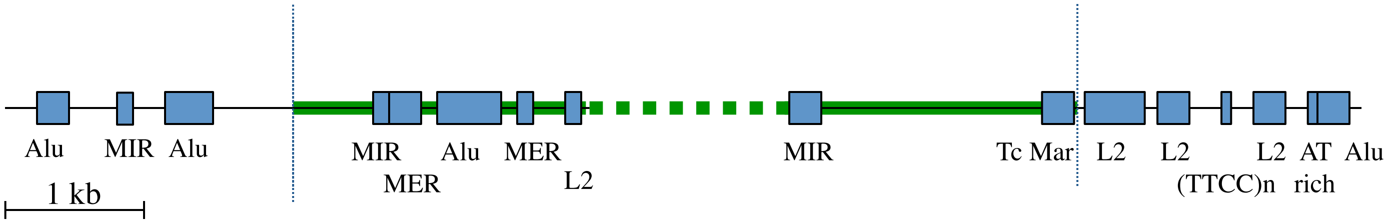

B. SW613-Tu1 junction between chromosome 17 and 7

```
                   chr 17 (53.497.490) - chr 7 (23.542.171)
                         strand -               strand -

AATTGCCCCTTCCTTGTTTGATTAAGAGTTTGTCATTCTTACCTATACCTTTGGATCCTCTAAAAGAAAAAAAA
||||||||||||||||||||||||||||||||||||||
AATTGCCCCTTCCTTGTTTGATTAAGAGTTTGTCATTCAGGTGTGAGCCACCACGTCCAGCAGAGTATTTGGCTT
                                     ||||||||||||||||||||||||||||||||||||||
AATTGACCTCCCTCAGCCTCGAAAAGTGTTGGGATTACAGGTGTGAGCCACCACGTCCAGCAGAGTATTTGGCTT
```

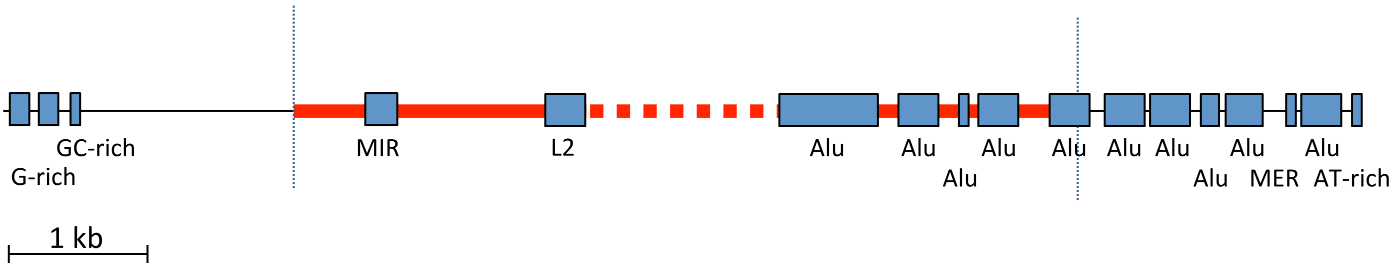

C. SW613-TU1 junction of the chromosome 17 deletion

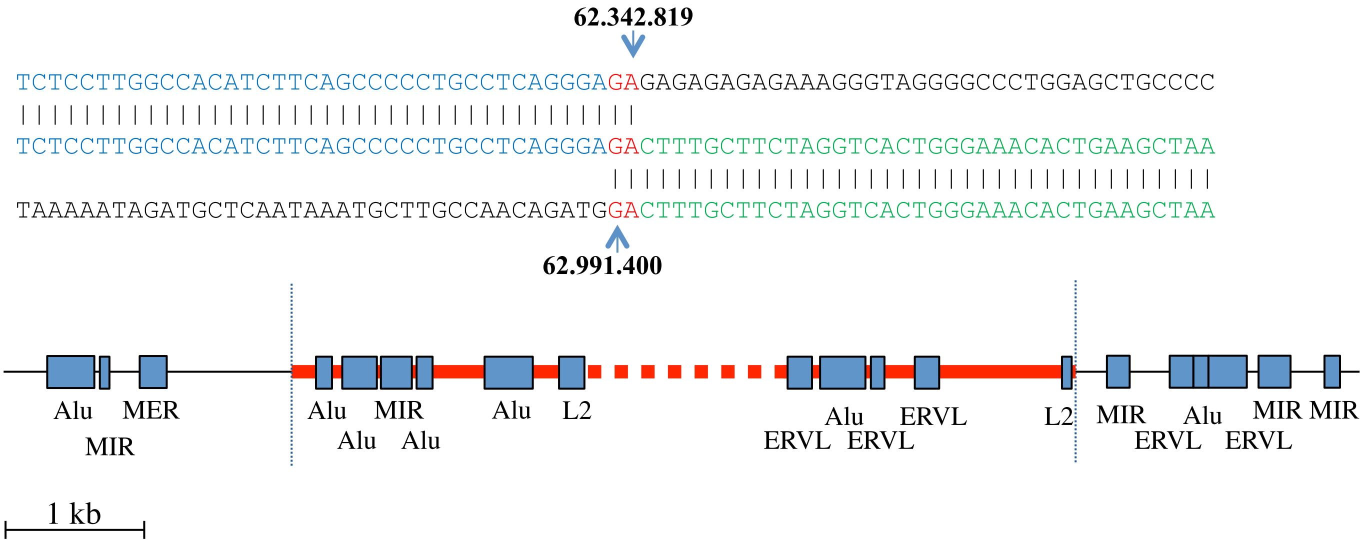

D. SW613-Tu1 head to head chromosome 17 junction

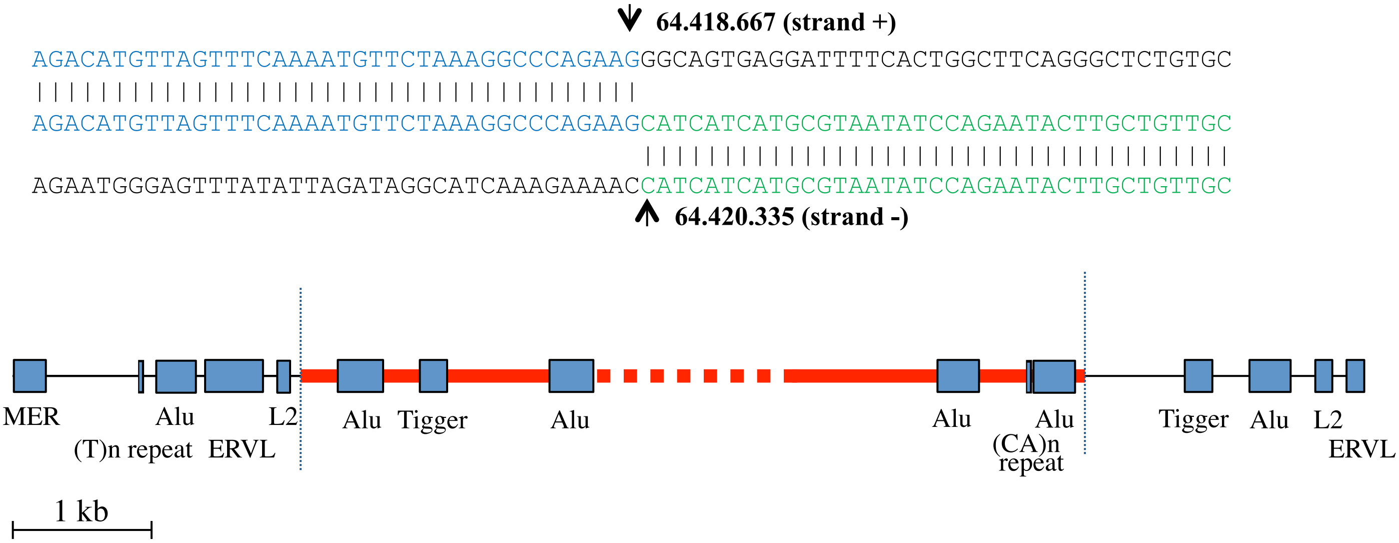

E. SW613-Tu1 junction between chromosomes 17 and 21

```
                                      ↓ 63.579.653 (chr17 strand -)
ACCATCCTGGCCAACCTGGTGAAACCCCATCTCTACTAAAAACACACAAAAATTAGCAAGTTGTGGTGTCAGACA
|||||||||||||||||||||||||||||||||||||||
ACCATCCTGGCCAACCTGGTGAAACCCCATCTCTACTAAGGGCCTGGCTAATTTTTTGTATTTTTAGTAGAGACA
                                         ||||||||||||||||||||||||||||||||||
CAGCCTCCCGAGCAGCTGGGACTGCAGGCGCCCGCCACCAAGCCTGGCTAATTTTTTGTATTTTTAGTAGAGACA
                                         ↑31.154.562 (chr21 strand +)
```

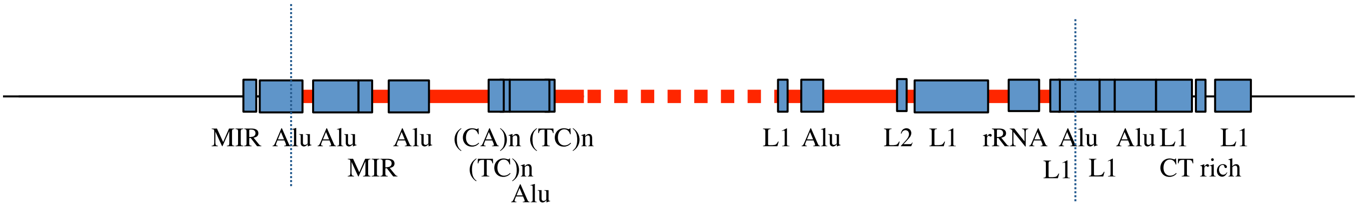

F. SW613-Tu1 junction between chromosomes 8 and 21

```
                                       ↓31.154.717 (chr21 strand +)
CAGGCCACTTTTTTCGGTAATGTTCCCTTTCTTTGGGACATATGTCAGGAATGGAATTGCTGAGTCATGGAGAAA
|||||||||||||||||||||||||||||||||||||||
CAGGCCACTTTTTTCGGTAATGTTCCCTTTCTTTGGGACGGGGGTGCATGGAGGGTGAGGATCAAAAAACTACCT
                                       ||||||||||||||||||||||||||||||||||||
AAGAAGGGATCTGCAGATACCAGGGCCTACTGGAGGGTGGGGGGTGCATGGAGGGTGAGGATCAAAAAACTACCT
                                       ↑127.519.888 (chr8 strand +)
```

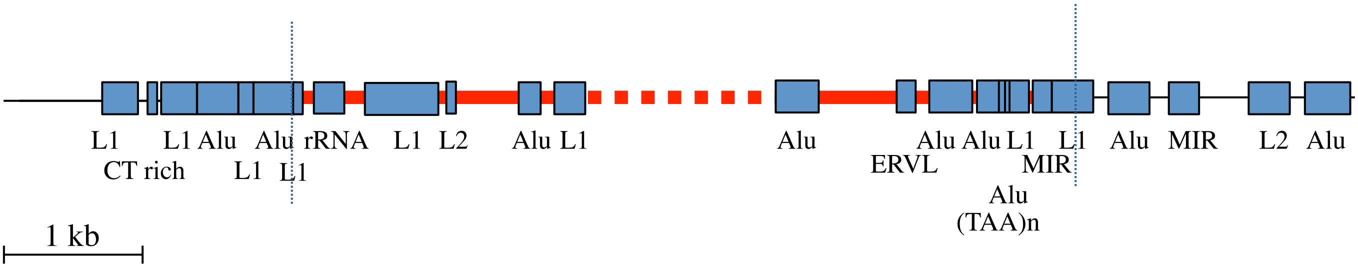

Figure S4

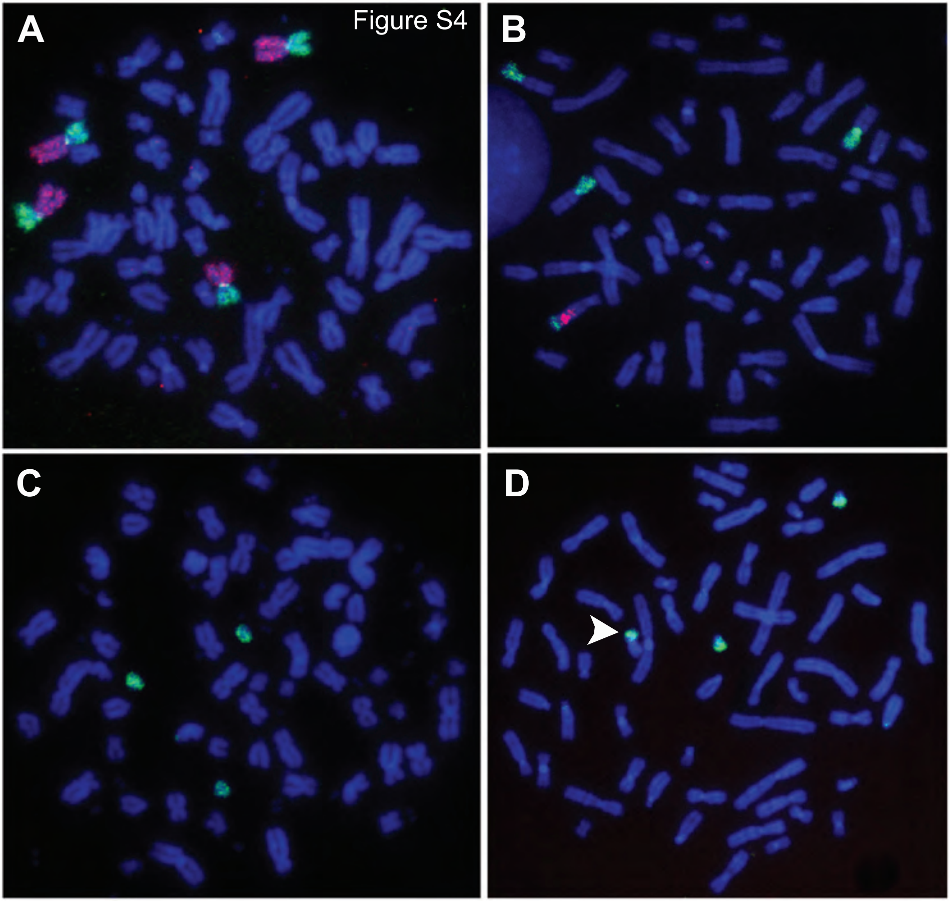

Figure S5

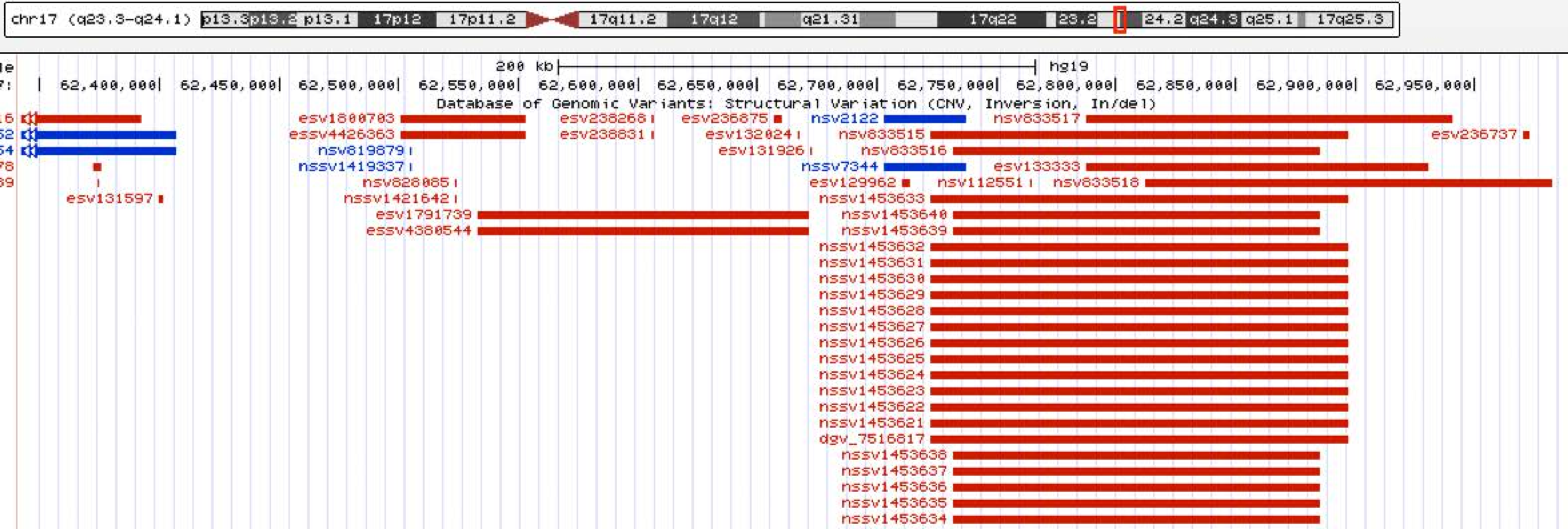
chr17 (q23.3-q24.1)
Scale
200 kb
hg19
chr17:
62,400,000
62,450,000
62,500,000
62,550,000
62,600,000
62,650,000
62,700,000
62,750,000
62,800,000
62,850,000
62,900,000
62,950,000
Database of Genomic Variants: Structural Variation (CNV, Inversion, In/del)
esv132616
nsv524852
nssv700854
nsv112578
esv131989
esv131597
esv1800703
essv4426363
nsv819879
nssv1419337
nsv828065
nssv1421642
esv1791739
essv4380544
esv238268
esv238631
esv236675
esv132024
esv131926
nsv2122
nsv833515
nsv833516
nssv7344
esv129962
nsv112551
nsv833517
esv133333
nsv833518
esv236737
nssv1453633
nssv1453640
nssv1453639
nssv1453632
nssv1453631
nssv1453630
nssv1453629
nssv1453628
nssv1453627
nssv1453626
nssv1453625
nssv1453624
nssv1453623
nssv1453622
nssv1453621
dgv_7516817
nssv1453638
nssv1453637
nssv1453636
nssv1453635
nssv1453634

Figure S6

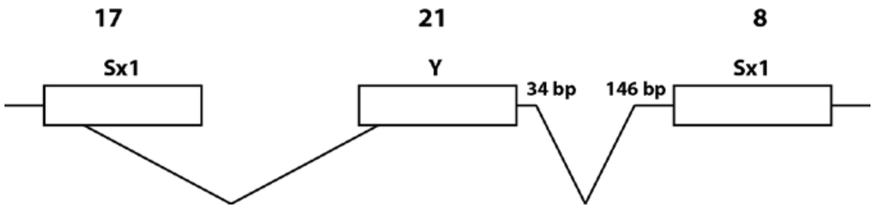

Chromosome	Alu			Junction	
	Type	Start	End	5'	3'
17	Sx1	63,579,736	63,579,472		63,579,653
21	Y	31,154,403	31,154,683	31,154,562	31,154,717
8	Sx1	127,520,134	127,520,444	127,519,888	

Supporting information. Legend

Supporting figure S1

GBM11. Structure of the amplicon. **A**. Sequences of the junction of the amplicon. The sequence was aligned with respect to the sequences of its two normal chromosome 8 counterparts (5' counterpart above the junction and 3' counterpart below it). Microhomology sequences are shown in red. **B**. Repetitive sequences in the vicinity of the breakpoints (modified from the human genome sequence at the UCSC Genome Bioinformatics site). The repetitive sequences present in the 2 kb on both sides of the junction are displayed. The repeat elements are boxed. The names of the sequences are specified below each box. L: LINE element; LTR: long terminal repeat; MaLR: retrovirus-like element; Mariner: mobile element; MER: medium reiteration frequency repetitive sequence; MIR: medium interspersed frequency repetitive sequence. Thick green lines correspond to the amplified fragment and thin lines to non-amplified sequences. The dotted vertical lines locate the breakpoint/fusion positions

Supporting data S1

GBM11. Localization of the hsr insertion site by FISH.

Supporting figure S2

GBM11: Structure of the site of insertion of the hsr. **A**. Junction positions and sequences. The sequence of each junction is aligned with respect to the sequences of its two normal counterparts. Sequences from chromosome 17 are in blue. Sequences from the chromosome 8 amplicon present in the hsr are in green. The

chromosome 17 deleted segment is in red. **B**. Repetitive sequences in the vicinity of the breakpoints (modified from the human genome sequence at the UCSC Genome Bioinformatics site). The repetitive sequences present in the 2 kb on both sides of the junction are displayed. The repeat elements are boxed. The names of the sequences are specified below each box; L: LINE element; MaLR: retrovirus-like element; MER: medium reiteration frequency repetitive sequence; MIR: medium interspersed frequency repetitive sequence. Thick green lines correspond to the amplified fragment and thin lines to non-amplified sequences. The dotted vertical lines locate the breakpoint/fusion positions

Supporting data S2

GBM11. Rearrangements of chromosome 17.

Supporting figure S3

Tumours SW613-3 and SW613-Tu1. Structure of the junctions and repetitive sequences in the vicinity of the breakpoints. **A**. Junction of the MYC amplicon. **B**. SW613-Tu1: junction between chromosome 17 and 7. **C**. SW613-Tu1 chromosome 17 deletion. **D**. SW613-Tu1-1: head-to-head chromosome 17 junction. **E**. SW613-Tu1: junctions between chromosomes 17 and 21. The sequences are aligned with respect to the sequences of its two normal counterparts. Repetitive sequences are modified from the human genome sequence at the UCSC Genome Bioinformatics site. Repetitive sequences in the vicinity of the breakpoints (modified from the human genome sequence at the UCSC Genome Bioinformatics site). The repetitive sequences present in the 2 kb on both sides of the junction are displayed. The repeat elements are boxed. The names of the sequences are specified below each box. Alu:

repetitive Alu element; ERVL: endogenous retrovirus class III; L: LINE element; Mariner: mobile element; MER: medium reiteration frequency repetitive sequence; MIR: medium interspersed frequency repetitive sequence; Tigger: Transposon.The dotted vertical lines locate the breakpoint/fusion position. Microhomologies and inserted sequences are shown in red. Thick green and red lines correspond to amplified and deleted fragments, respectively.

Supporting figure S4

A. SW613-3. Co-hybridization of the chromosome 7p (green) and 7q (red) paintings. Four normal chromosomes are labeled.

B. SW613-Tu1. Co-hybridization of the chromosome 7p painting (green) and of the BAC RP11-1136L8 containing *MYC* (red). Three normal chromosomes 7 and the distal part of the rearranged chromosome containing the hsr are labelled by the painting.

C. SW613-3. Hybridization of chromosome 21 painting (green). Three normal chromosomes are labeled.

D. SW613-Tu1. Hybridization of chromosome 21 painting (green). Two normal chromosomes and one translocated chromosome ((arrowhead) are labelled.

Supporting figure S5

Deleted region in the chromosome 17. Locaization of copy number variants, blue and red correspond to gain and loss in size relative to the reference. From the UCSC genome browser on human Feb 2009 (GRCh37/hg9) assembly.

Supporting figure S6

SW613-Tu1. Position of the junctions between segments from chromosome 17, 21 and 8 at the site of insertion of the hsr in relationship to the position of the Alu repeats. The table gives the positions of the Alu repeats and of the junctions.
